# Vasorelaxant properties of *Vernonia amygdalina* ethanol extract and its possible mechanism

**DOI:** 10.1080/13880209.2017.1357735

**Published:** 2017-08-23

**Authors:** Yung Sing Ch’ng, Yean Chun Loh, Chu Shan Tan, Mariam Ahmad, Mohd. Zaini Asmawi, Wan Maznah Wan Omar, Mun Fei Yam

**Affiliations:** a School of Pharmaceutical Sciences, Universiti Sains Malaysia, Pulau Pinang, Malaysia;; b School of Biological Sciences, Universiti Sains Malaysia, Pulau Pinang, Malaysia

**Keywords:** Tri-step FTIR, calcium channel, potassium channel, NO/cGMP pathway, prostacyclin

## Abstract

**Context:***Vernonia amygdalina* Del. (VA) (Asteraceae) is commonly used to treat hypertension in Malaysia.

**Objective:** This study investigates the vasorelaxant mechanism of VA ethanol extract (VAE) and analyzes its tri-step FTIR spectroscopy fingerprint.

**Materials and methods:** Dried VA leaves were extracted with ethanol through maceration and concentrated using rotary evaporator before freeze-dried. The vasorelaxant activity and the underlying mechanisms of VAE using the cumulative concentration (0.01–2.55 mg/mL at 20-min intervals) were evaluated on aortic rings isolated from Sprague Dawley rats in the presence of antagonists.

**Results:** The tri-step FTIR spectroscopy showed that VAE contains alkaloids, flavonoids, and saponins. VAE caused the relaxation of pre-contracted aortic rings in the presence and absence of endothelium with EC_50_ of 0.057 ± 0.006 and 0.430 ± 0.196 mg/mL, respectively. In the presence of Nω-nitro-l-arginine methyl ester (EC_50_ 0.971 ± 0.459 mg/mL), methylene blue (EC_50_ 1.203 ± 0.426 mg/mL), indomethacin (EC_50_ 2.128 ± 1.218 mg/mL), atropine (EC_50_ 0.470 ± 0.325 mg/mL), and propranolol (EC_50_ 0.314 ± 0.032 mg/mL), relaxation stimulated by VAE was significantly reduced. VAE acted on potassium channels, with its vasorelaxation effects significantly reduced by tetraethylammonium, 4-aminopyridine, barium chloride, and glibenclamide (EC_50_ 0.548 ± 0.184, 0.158 ± 0.012, 0.847 ± 0.342, and 0.304 ± 0.075 mg/mL, respectively). VAE was also found to be active in reducing Ca^2+^ released from the sarcoplasmic reticulum and blocking calcium channels.

**Conclusions:** The vasorelaxation effect of VAE involves upregulation of NO/cGMP and PGI_2_ signalling pathways, and modulation of calcium/potassium channels, and muscarinic and β_2_-adrenergic receptor levels.

## Introduction

Hypertension is the most common risk factor of cardiovascular diseases. It is an important public health problem worldwide due to its high prevalence and its detrimental effects such as stroke, chronic kidney disease, and cardiac restructuring and damage (Rampal et al. 2008). According to the Hypertension Clinical Practice Guidelines (CPG) of Malaysia on 2013, the prevalence of citizens aged 18  years old and above who are suffering from hypertension has increased from 32.2% in 2006 to 32.7% in 2011, whereas the prevalence of those aged 30  years old and above has increased from 42.6 to 43.5% (Rahman 2013). Therefore, there is an imminent need to decrease the prevalence of hypertension. Although lowering blood pressure by relaxing arteries can greatly reduce hypertension complication such as cerebrovascular, cardiovascular, and renal disease (Wang et al. 2014), the common antihypertensive drugs present in the market nowadays have low effectiveness and undesired chronic side effects. Thus, seeking new effective antihypertensive drugs with minimal side effects is urgent for human populations. Currently, there is a growing awareness in several countries on the importance of native plant remedies in the healthcare delivery system due to the biodiversity of native plants. Efforts are concentrated on investigating the therapeutic efficacy of locally available medicinal herbs because they are more readily available and are perceived to be safer (Taiwo et al. 2010).

*Vernonia amygdalina* Del. (VA) (Asteraceae), commonly known as ‘African bitter leaf’ or ‘bitter leaf’, is a plant that grows in the tropical areas of Africa, particularly in Nigeria (Atangwho, Ebong, et al. 2012; Atangwho, Edet, et al. 2012; Agbogidi and Akpomorine 2013). In Africa, it is a multipurpose vegetable that can be used as food and as a traditional treatment for diseases, such as tonsillitis, fever, malaria, diabetes, pneumonia, jaundice, anaemia, stomach problems, and ascariasis (Quasie et al. 2016). This plant was introduced and it is currently growing wild in Malaysia and is being used as a remedy for the management of diabetes mellitus and hypertension (Atangwho et al. 2013). Previous studies reported that VA is capable of effects such as antioxidant, antidiabetic, anti-obesity, and antihypertensive (Taiwo et al. 2010; Atangwho, Edet, et al. 2012; Atangwho et al. 2013; Olaiya et al. 2013). It is known that the blood pressure lowering effect of VA is mediated through vasorelaxation (Taiwo et al. 2010). However, the vasorelaxant mechanisms of VA have not been clearly studied.

Our previous study (Ch’ng et al. 2016) found that the ethanol extract of VA (VAE) showed the greatest vasorelaxant activity. Therefore, this study determines the possible vasorelaxation mechanism of VAE through vascular reactivity experiments. The fingerprint of VAE was also analyzed using the tri-step Fourier transform infrared (FTIR) method consisting of conventional FTIR, second derivative infrared (SD-IR), and two-dimensional correlation infrared (2D-correlation IR) as references for quality management to obtain similar pharmacodynamic effects.

## Materials and methods

### Materials

Acetylcholine chloride (ACh), phenylephrine hydrochloride (PE), and nifedipine were purchased from Acros Organics (Geel, Belgium). N_ω_-nitro-l-arginine methyl ester (L-NAME), indomethacin, tetraethylammonium chloride (TEA), barium chloride (BaCl_2_), glibenclamide, 2-aminoethyl diphenylborinate (2-APB), 1H-(1,2,4)oxadiazolo(4,3-a)quinoxalin-1-one (ODQ), atropine, and propranolol hydrochloride were purchased from Sigma-Aldrich (St. Louis, MO). Ethylene glycol-bis(2-aminoethylether)-*N*,*N*,*N*′,*N*′-tetraacetic acid (EGTA) was purchased from Calbiochem (Darmstadt, Germany), and methylene blue (MB) was purchased from Promedipharm Sdn. Bhd. (Selangor, Malaysia). 4-Aminopyridine (4-AP) and potassium bromide (KBr) were purchased from Merck (Darmstadt, Germany).

### Experimental animals

Adult male Sprague Dawley (SD) rats weighing 250–300 g were obtained from the Animal House at Universiti Sains Malaysia. The rats were housed at room temperature with a 12-h light/dark cycle and allowed free access to feed and water. The investigation conforms to the Guide for the Care and Use of Laboratory Animal by Universiti Sains Malaysia and was approved by the Animal Ethical Committee of the School of Pharmaceutical Sciences, Universiti Sains Malaysia [USM/Animal Ethics Approval/2016/(103) (771)].

### Plant material and extraction

Fresh leaves of VA were collected from Paya Terubong, Penang, Malaysia (January–March 2015). The plant was identified by Dr. Rahmad Zakaria and a voucher specimen 11706 was deposited in the Herbarium of the School of Biological Sciences, Universiti Sains Malaysia. The leaves were washed with tap water and dried in an oven at 50 °C. The dried leaves were ground into fine powder and extracted with ethanol by maceration at 55 °C for 48 h. After that, VAE was concentrated in a rotary evaporator under vacuum and lyophilized using a freeze drier. The lyophilized extract was stored in the refrigerator at 4 °C to prevent any decomposition of the components.

### Fingerprint analysis by tri-step FTIR spectroscopy

The dry VAE (around 1–2 mg) was mixed evenly with 100 mg of KBr crystal. The mixture was then pressed into a tablet with a pressure of not more than 10 psi. The spectra were obtained under the conditions of 16 co-added scans in the range of 400–4000 cm^−1^ with a resolution of 4 cm^−1^. Before scanning the sample, a tablet of pure KBr was scanned as background, and then the interferences of atmospheric water and carbon dioxide were eliminated online during the scanning of the sample. The tri-step Fourier transform infrared (FTIR) spectrum of the sample was generally accepted when a transmission higher than 60% was achieved. Otherwise, the test had to be repeated with either the addition of sample or KBr (Li et al. 2010; Li et al. 2014).

The second derivative infrared (SD-IR) spectrum of VAE was obtained after Savitzky–Golay polynomial fitting (13-point smoothing) of the original IR spectrum taken at room temperature. To obtain the two-dimensional correlation infrared (2D-IR) spectra, the sample tablet was placed into the sample holder with a programmable heated jacket controller (Model GS20730; Specac, Orpington, England). In order to avoid the loss or change of some unstable compositions, the dynamic spectra were collected at various temperatures ranging from room temperature to 120 °C at an interval of 10 °C with a heating rate of 2 °C/min. The 2D-IR correlation spectra were then obtained by treating a series of dynamic spectra with a 2D-IR correlation analysis software developed by Tsinghua University, Beijing, China (Li et al. 2010; Li et al. 2014).

### Preparation of blockers and extract

ACh (1 µM), PE (1 µM), L-NAME (10 µM), MB (10 µM), TEA (1 mM), 4-AP (1 mM), BaCl_2_ (10 µM), propranolol (1 µM), and atropine (1 µM) were prepared in distilled water (Rameshrad et al. 2016). Indomethacin (10 µM), nifedipine (1 µM), 2-APB (100 µM), ODQ (1 µM), and glibenclamide (10 µM) were dissolved with 1% Tween 80. VAE was prepared in 1% Tween 80 at a concentration of 128 mg/mL as the stock solution. The stock solution was then diluted to working concentration with distilled water.

### Aortic ring preparation

The contractile responses of the aortic smooth muscle of male SD rat to VAE were determined through an *in vitro* study. First, the SD rat was anesthetized by carbon dioxide inhalation before isolation of its aorta. The aorta was then transferred to a Petri dish filled with Krebs–Henseleit (Krebs) solution (118.0 mM NaCl, 4.7 mM KCl, 25.0 mM NaHCO_3_, 1.8 mM CaCl_2_, 1.2 mM NaH_2_PO_4_, 1.2 mM MgSO_4_, and 11.0 mM glucose) while the connective tissues around the aorta were removed. During the process, the Krebs solution containing the aorta was continuously supplied with ‘carbogen’ (95% O_2_ and 5% CO_2_). After the aorta was cleaned, it was cut into 2–3 mm aortic ring segments and suspended horizontally in an organ bath that contains 10 mL of Krebs solution supplied constantly with carbogen gas at 37 °C. Special care was taken to avoid damage to the endothelium. When preparing endothelium-denuded aorta, the endothelium was removed with a cotton swab by gently rubbing the internal space of the aorta (Wang et al. 2014). The aortic rings were allowed to equilibrate at an optimal tension of 1 g for 30 min. Throughout this period, the Krebs solution was replaced every 10 min and the tension was readjusted to 1 g if needed. For confirmation of the condition of the aortic rings, they were pre-contracted with PE (1 µM) followed by relaxation with Ach (1 µM). After that, the rings were rinsed with Krebs solution and the tension was readjusted to 1 g, and 1 µM of PE was added to establish a stable contractile tone. When a plateau was achieved, a concentration–response curve was constructed by the cumulative addition of the VAE (0.01, 0.02, 0.04, 0.08, 0.16, 0.32, 0.64, and 1.28 mg/mL) at 20-min intervals on endothelium-denuded and endothelium-intact rings (Ameer et al. 2010; Loh et al. 2017; Tan, Ch’ng, et al. 2017; Tan, Loh, et al. 2017).

Changes of the contractile force on the aortic rings were measured with a force-electricity transducer (GRASS Force-Displacement Transducer FT03 C Isometric Force Measurements). Signals were amplified, generated by the LabChart 5 software, and the data were tabulated by using Microsoft Excel.

### Determination of the effects of VAE on cGMP-coupled signal transduction and prostacyclin signalling pathway

To determine the effects of VAE on nitric oxide (NO), cyclic guanosine monophosphate (cGMP), and prostacyclin (PGI_2_), the endothelium-intact aortic rings were incubated with the NO synthase inhibitor, L-NAME (10 µM), PGI_2_ synthesis inhibitor, indomethacin (10 µM), soluble guanylyl cyclase (sGC) inhibitor, ODQ (1 µM), and cGMP lowering agent, methylene blue (10 µM) for 20 min prior to pre-contraction by PE (Tan, Ch’ng, et al. 2017). Comparisons were made between the cumulative concentration–response of VAE to the aortic rings with and without pre-incubation with the above inhibitors.

### Determination of the roles of VAE on muscarinic andβ_2_-adrenergic receptors

To investigate the role of VAE on muscarinic receptors and β_2_-adrenergic receptors, endothelium-intact aortic rings were incubated with muscarinic receptor antagonist, atropine (1 µM) and β_2_-adrenergic antagonist, propranolol (1 µM) for 20 min prior to pre-contraction with PE (1 µM) (Tan, Ch’ng, et al. 2017). A comparison was made between the cumulative concentration response of VAE on aortic rings with and without pre-incubation with the abovementioned inhibitors.

### Determination of the effects of VAE on potassium channels

To determine the effects of VAE on potassium (K^+^) channels, the nonselective calcium-activated K^+^ channel (K_Ca_) blocker, TEA (1 mM), voltage-dependent K^+^ channel (K_v_) blocker, 4-AP (1 mM), inwardly rectifying K^+^ channel (K_ir_) blocker, BaCl_2_ (10 μM), or nonspecific ATP-sensitive K^+^ channel (K_ATP_) blocker, glibenclamide (10 μM) was applied to the aortic rings (endothelium intact) for 20 min prior to pre-contraction by PE (Senejoux et al. 2010; Tan, Ch’ng, et al. 2017). The results obtained were compared between the cumulative concentration response of VAE in aortic rings with and without pre-incubation with the blockers mentioned above.

### Determination of the effects of VAE on calcium-induced vasoconstriction

To determine the effects of VAE on L-type calcium channels, three sets of experiments were carried out, namely the control, nifedipine, and VAE groups. For the control group, the aortic rings (endothelium-intact) were allowed to stabilize in normal Krebs solution for 30 min, which was then replaced with Ca^2+^-free Krebs solution containing EGTA (0.2 mM) for 20 min (by washing and replacing the solution in the organ bath twice, at 10-min interval each) in order to remove Ca^2+^ from the tissues. The aortic rings were then rinsed in a Ca^2+^-free Krebs solution (without EGTA) for 20 min (by washing and replacing the solution in the organ bath twice, at 10-min interval each). Then, the Ca^2+^ (0.1, 0.3, 1.0, 3.0, 10.0, 30.0, and 100.0 µM) was added cumulatively into the organ bath at 3-min intervals. For the nifedipine group, nifedipine (0.1, 0.3, and 1 µM) was applied to the aortic rings (endothelium-intact) for 20 min prior to the cumulative additions of Ca^2+^ (0.1, 0.3, 1.0, 3.0, 10.0, 30.0, and 100.0 µM) to the aortic rings at 3-min intervals. The experiment for the VAE group was carried out in a similar way to the nifedipine group but with the aortic rings pre-incubated with VAE (0.01, 0.04, 0.16, and 0.64 mg/mL) instead of nifedipine before being contracted with Ca^2+^ (Ameer et al. 2010; Wang et al. 2010; Senejoux et al. 2013; Tan, Ch’ng, et al. 2017).

### Determination of the effects of VAE on intracellular Ca^2+^ release from sarcoplasmic reticulum

The experiment was conducted to determine the relaxation effect of VAE on the inhibition of intracellular Ca^2+^ release. Endothelium-denuded aortic rings were allowed to stabilize in a Ca^2+^-free Krebs solution for 20 min. The Krebs solution was replaced with EGTA (0.2 mM) and Ca^2+^-free Krebs solution for 10 min. VAE (0.01, 0.04, 0.16, and 0.64 mg/mL) or 2-APB (100 µM) was used to pre-incubate the aortic rings for 20 min before PE (1 µM) was added. The group without incubation with VAE was considered to be the control (Tan, Ch’ng, et al. 2017).

### Statistical analysis

All results were expressed as mean ± SEM. Statistical analysis was performed by one-way ANOVA using the SPSS version 20 software (SPSS Inc., Chicago, IL). *p*-Values less than 0.05 were considered significant in all cases.

## Results

### Tri-step IR macro-fingerprint of VAE

**Figure 1.**
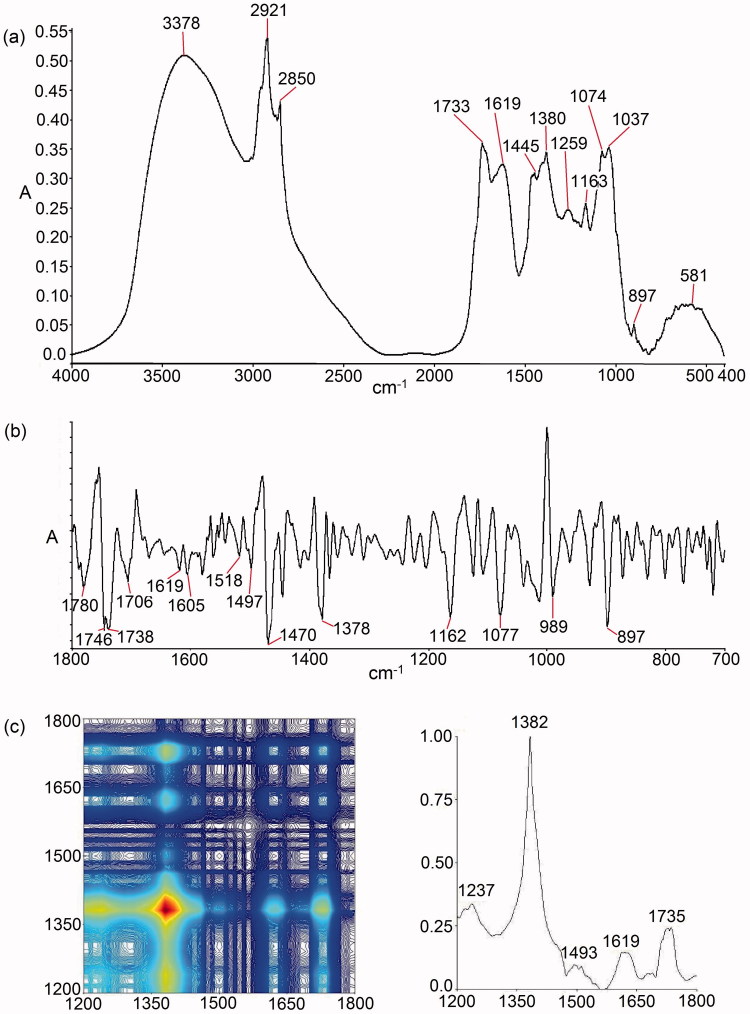
Fingerprint of VAE in (a) conventional FTIR, (b) second derivative in the range of 1800–700 cm^−1^, and (c) 2D-correlation IR spectra in the range of 1200–1800 cm^−1^.

Figure 1 shows the fingerprint of VAE generated by using the tri-step Fourier transform infrared (FTIR) method. In the conventional FTIR spectrum of VAE (Figure 1(a)), characteristic peaks were located at 3378, 2921, 2850, 1733, 1619, 1445, 1380, 1259, 1163, 1074, 1037, 897, and 581 cm^−1^. The assignment of these peaks is shown in Table 1. The second derivative infrared (SD-IR) spectrum of VAE in the range of 1800–700 cm^−1^ is shown in Figure 1(b). The resolution of the original IR spectrum has been enhanced to resolve the problem of overlapping peaks. Therefore, some characteristic peaks hidden in the original IR spectrum can be clearly seen in its SD-IR spectrum, such as those at 1780, 1746, 1706, 1470, 1605, 1518, 1497, and 989 cm^−1^, whereas the synchronous 2D-correlation IR spectra of VAE in the range of 1200–1800 cm^−1^ had showed its auto peaks at 1237, 1382, 1493, 1619, and 1735 cm^−1^. These auto peaks form 5 × 5 peak clusters and their corresponding cross peaks are positive (Figure 1(c)).

**Table 1.** Peak assignments on the FTIR spectrum of VAE.

Peak (cm^−1^)		
VAE	Primary assignment	Possible compounds
3378	O–H and N–H, ν	Various and alkaloid
2921	C–H, ν_as_	Various
2850	C–H, ν_s_	Various
1733	C = O, ν	Ester
1619	O–H, δ and Ring	Ester and aromatic
1445	Ring	Aromatic
1380	C–H, δ	–
1259	(O) C–H, δ	Saccharides
1163	C–O, ν	Saccharides
1074	C–O, ν	Saccharides
1037	C–O, ν	Saccharides

ν: stretching; ν_s_: symmetrical stretching; ν_as_: asymmetrical stretching; δ: bending.

### Effects of VAE on PE pre-contracted aortic rings

**Figure 2.**
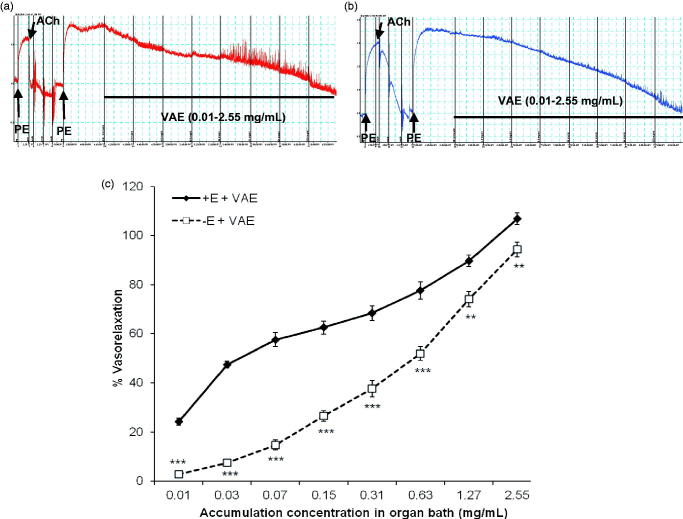
Original isometric force recordings showing the contraction evoked by PE in endothelium-intact (a) and endothelium-denuded (b) rat aortic rings treated with various concentrations of VAE. Effect of VAE on PE-induced contraction in endothelium-intact aortic rings and endothelium-denuded aortic rings (*n* = 8) (c). *, **, and *** indicate significance at *p* < 0.05, *p* < 0.01, and *p* < 0.001, respectively, compared to the group of endothelium-intact aortic rings.

By looking at the results on Figure 2, the effect of VAE manifested in a concentration-dependent manner in both endothelium-intact and endothelium-denuded aortic rings. VAE was able to induce relaxation in the rat aortic rings with an EC_50_ of 0.057 ± 0.006 mg/mL (accumulative concentration) and the R_max_ was 106.91 ± 2.34% in endothelium-intact aortic rings. However, in endothelium-denuded aortic rings, the vasorelaxant effect of VAE was less prominent than in endothelium-intact aortic rings, with a 12.59% reduction and an EC_50_ value of 0.430 ± 0.196 mg/mL.

### Roles of VAE on endothelium-dependent vasorelaxant factors

**Figure 3.**
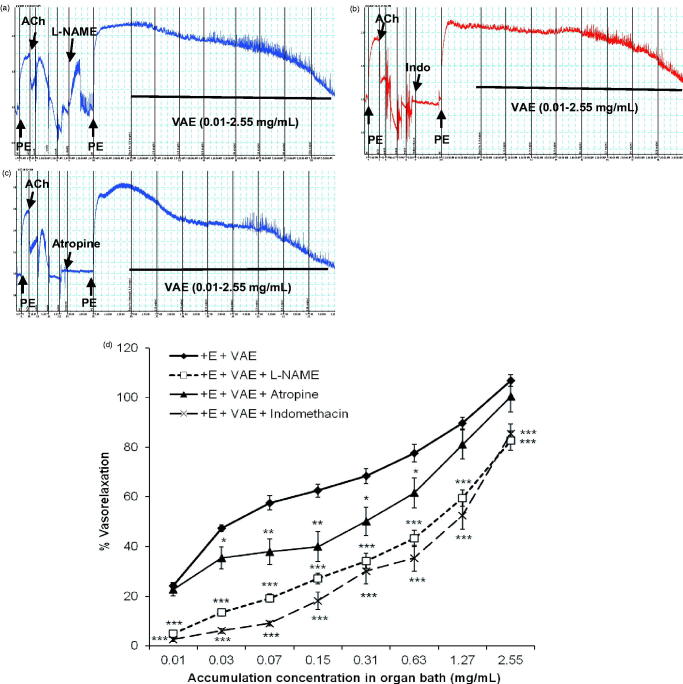
Original isometric force recordings showing influence of L-NAME (a), indomethacin (b), and atropine (c) on the vasorelaxant effect of VAE in endothelium-intact aortic rings. Effect of VAE on PE-induced contraction in endothelium-intact aortic rings (*n* = 8) in the presence of L-NAME, indomethacin, and atropine (d). *, **, and *** indicate significance at *p* < 0.05, *p* < 0.01, and *p* < 0.001, respectively, compared to the group of endothelium-intact aortic rings.

As shown in Figure 3, the inhibition of eNOS, PGI_2_, and muscarinic receptor pathways was associated with a significant decrease in vasorelaxant effect of VAE on PE-constricted aortic rings. The L-NAME and indomethacin significantly reduced vasorelaxation, with R_max_ values of 82.70 ± 3.97% and 85.52 ± 3.79% (*n* = 8, *p* < 0.001) as well as EC_50_ values of 0.971 ± 0.459 mg/mL and 2.128 ± 1.218 mg/mL. In contrast, atropine exhibited a mild inhibition effect on vasorelaxation, with an R_max_ of 100.42 ± 6.30% (*n* = 8, *p* > 0.05) and a slight increase in the EC_50_ values to 0.470 ± 0.325 mg/mL.

### Endothelium-independent mechanisms of VAE

**Figure 4.**
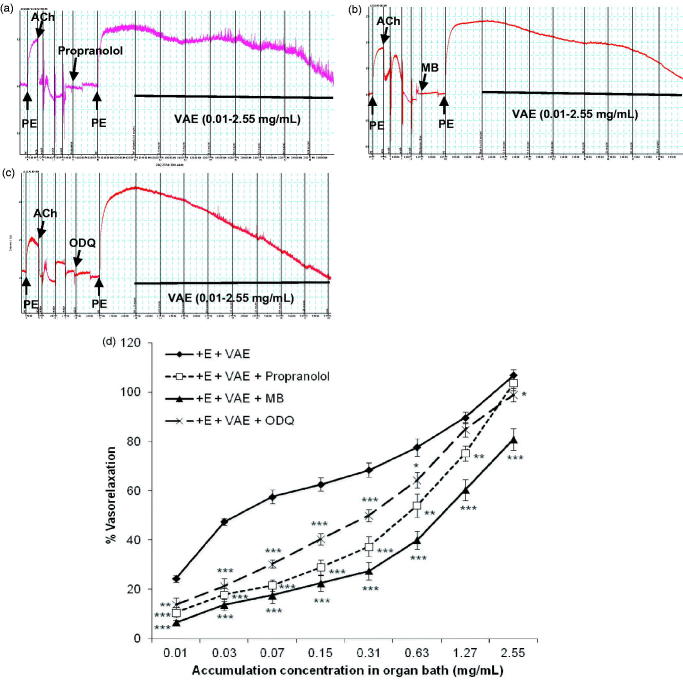
Original isometric force recordings showing influence of propranolol (a), methylene blue (b), and ODQ (c) on the vasorelaxant effect of VAE in endothelium-intact aortic rings. Effect of VAE on PE-induced contraction in endothelium-intact aortic rings (*n* = 8) in the presence of propranolol, methylene blue, and ODQ (d). *, **, and *** indicate significance at *p* < 0.05, *p* < 0.01, and *p* < 0.001, respectively, compared to the group of endothelium-intact aortic rings.

Figure 4 represents the effect of propranolol, methylene blue, and ODQ on vasorelaxant effect of VAE on contracted aortic rings. From Figure 4(d), pre-treatment with propranolol and ODQ significantly decreased the vasorelaxant effect of VAE during the application of the starting concentration (0.01–0.31 mg/mL) (*p* < 0.001). However, they were followed by a dramatic relaxation effect when a cumulative concentration up to 1.27 mg/mL (*p* < 0.01) was applied. Both of them subsequently ended up with almost the same amount of maximum relaxation compared to the control group at the final concentration, where the R_max_ values of the aortic rings pre-treatment with propranolol and ODQ are 103.77 ± 3.26% (*n* = 8, *p* > 0.05) and 98.94 ± 2.63% (*n* = 8; *p* < 0.05), respectively. The EC_50_ value obtained for VAE increased to 0.314 ± 0.032 mg/mL and 0.203 ± 0.027 mg/mL, respectively, when propranolol and ODQ are present. As for MB, it was found to amply reduce the vasorelaxant effect of VAE by 25.94% (R_max_ = 80.97 ± 4.39%, *n* = 8, *p* < 0.001) with an EC_50_ value of 1.203 ± 0.426 mg/mL as shown in Figure 4(d).

### Roles of VAE on potassium channels

**Figure 5.**
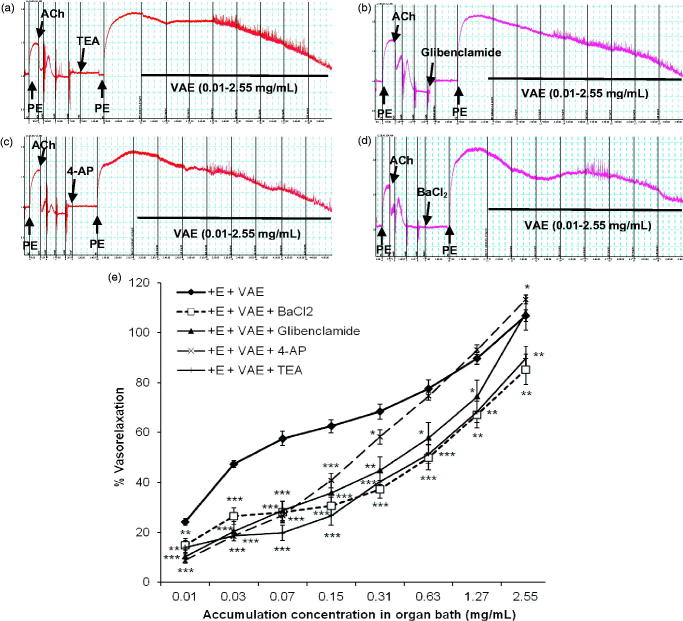
Original isometric force recordings showing the influence of TEA (a), glibenclamide (b), 4-AP (c), and BaCl_2_ (d) on the vasorelaxant effect of VAE in endothelium-intact aortic rings. Effect of VAE on PE-induced contraction in endothelium-intact aortic rings (*n* = 8) in the presence of TEA, glibenclamide, 4-AP, and BaCl_2_ (e). *, **, and *** indicate significance at *p* < 0.05, *p* < 0.01, and *p* < 0.001, respectively, compared to the group of endothelium-intact aortic rings.

Figure 5 represents the effect of potassium channel antagonists on vasorelaxant effect of VAE on contracted aortic rings. As seen in Figure 5(e), TEA largely inhibits the vasorelaxant effect exerted by VAE at 0.03–0.63 mg/mL (*p* < 0.001) but relaxation increased in the subsequent concentration applied. However, the maximum relaxation was still significantly reduced to R_max_ = 89.74 ± 4.84% (*n* = 8, *p* < 0.01), hence the EC_50_ value has increased to 0.548 ± 0.184 mg/mL. The selective blocker for K_ATP_, glibenclamide, produced a similar effect as TEA. From Figure 5(e), the starting cumulative concentration of VAE applied exhibited less relaxation effect compared to the control group (*p* < 0.001). But at concentration from 0.63 to 1.27 mg/mL, the vasorelaxant effect started to increase and the inhibition effect of glibenclamide was reduced (*p* < 0.05). The final concentration produced a R_max_ value of 108.06 ± 7.13% compared to the control group. However, the EC_50_ value increased to 0.304 ± 0.075 mg/mL. Pre-treatment with 4-AP also successfully suppressed the vasorelaxant effect of VAE at concentration from 0.01 to 0.31 mg/mL (*p* < 0.05), but a turning point occurred during the addition of a cumulative concentration of 0.63 mg/mL. The vasorelaxant effect exerted by VAE had increased beyond that of the control group (R_max_ = 113.31 ± 1.78%, *n* = 8, *p* < 0.05) with an EC_50_ value of 0.158 ± 0.012 mg/mL. For BaCl_2_, 10 µM of barium had an inhibition effect on the vasorelaxation properties elicited from VAE at the starting concentration of 0.01–0.63 mg/mL (*p* < 0.001). The vasorelaxant effect was slightly enhanced with a cumulative concentration of 1.27–2.55 mg/mL (*p* < 0.01). The R_max_ value was 85.26 ± 6.14% (*n* = 8), which was similar to the R_max_ value of indomethacin, and the EC_50_ value increased to 0.847 ± 0.342 mg/mL.

### Roles of VAE on calcium channels

**Figure 6.**
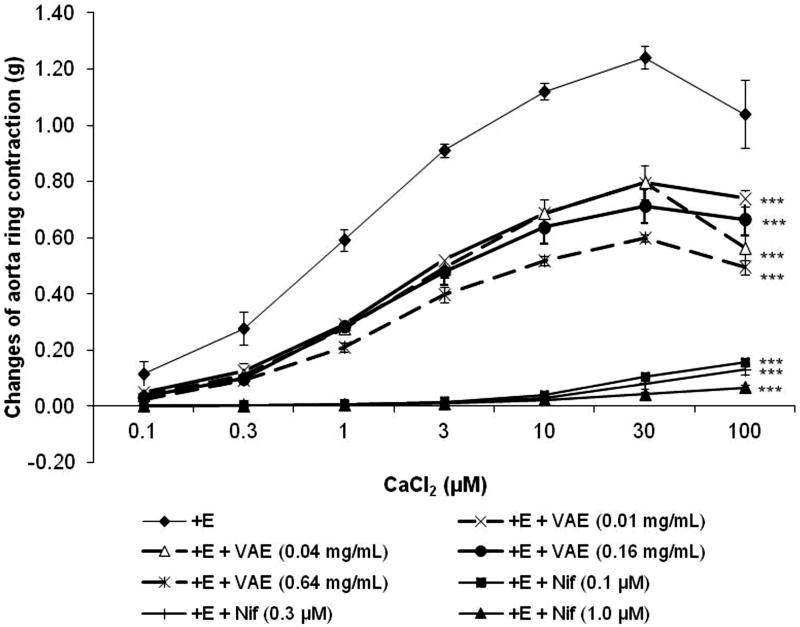
Effect of VAE on CaCl_2_-induced vasocontraction in isolated aortic rings (*n* = 8). *, **, and *** indicate significance at *p* < 0.05, *p* < 0.01, and *p* < 0.001, respectively, compared to the group without incubation of antagonist (control).

**Figure 7.**
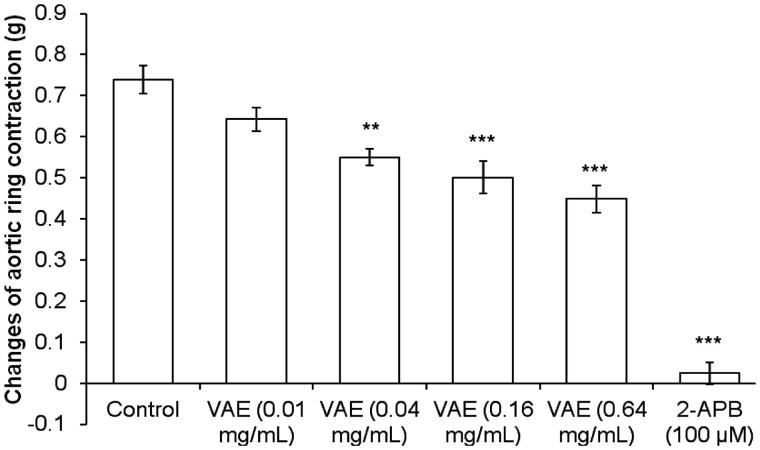
Vasorelaxant effect of VAE on PE pre-contracted endothelium-denuded aortic rings in Ca^2+^-free Krebs solution (*n* = 8). *, **, and *** indicate significance at *p* < 0.05, *p* < 0.01, and *p* < 0.001, respectively, compared to the control group.

From Figure 6, the contraction of the aortic rings induced by the cumulative addition of extracellular calcium (0.1–100 µM) into the organ bath showed a stepwise reduction. The results show that the contraction elicited by CaCl_2_ dramatically dropped to 0.16 ± 0.01 g (87% inhibition), 0.13 ± 0.01 g (90% inhibition), and 0.07 ± 0.01 g (94% inhibition) in the presence of 0.1 µM, 0.3 µM, and 1.0 µM (*n* = 8, *p* < 0.001) nifedipine compared to the control group that had a maximum contraction of 1.24 ± 0.04 g, whereas, in Figure 6, accounting for the differences in VAE concentrations (0.01, 0.04, 0.16, and 0.64 mg/mL), there was an attenuated contraction effect compared to the control group. A 35% inhibition of contraction was exhibited in the presence of VAE at concentrations 0.01 mg/mL (*n* = 8, *p* < 0.001) and 0.04 mg/mL (*n* = 8, *p* < 0.001), with a maximum contraction of 0.80 ± 0.01 g, whereas, with an increasing concentration of VAE to 0.16 mg/mL, the contraction effect was attenuated to 0.71 ± 0.06 g (*n* = 8, *p* < 0.001), and more than 50% of contraction was abolished in the presence of 0.64 mg/mL of VAE (0.60 ± 0.01 g, *n* = 8, *p* < 0.001). In the intracellular calcium release study, PE induced a transient contraction of 0.74 ± 0.03 g due to the release of Ca^2+^ from the sarcoplasmic reticulum. Pre-incubation of the aortic rings with VAE at concentrations of 0.04, 0.16, and 0.64 mg/mL significantly attenuated PE-induced contraction to 0.55 ± 0.02, 0.50 ± 0.04, and 0.45 ± 0.03 g, respectively (*p* *<* 0.01, 0.001, and 0.001). 2-APB at 100 µM also significantly decreased the vasoconstriction effect to 0.02 ± 0.03 g (*p* < 0.001) (Figure 7).

## Discussion

### Assignments of tri-step IR macro-fingerprint of VAE

From the conventional FTIR spectrum of VAE, the broad absorption peak at 3378 cm^−1^ was the stretching vibration of O–H bond and N–H bond. Its relatively sharp front shape indicated that VAE contains more N–H components. Coupled with the presence of peaks at 1619 cm^−1^ (C = O stretching vibration), 1445 cm^−1^ (aromatic ring skeleton vibration), and 1380 cm^−1^ (C–H bending vibration), this suggests that VAE contains alkaloids and flavonoids. In addition, the spectrum of VAE shows a strong peak at 1733 cm^−1^, indicating that VAE also contains esters. Meanwhile, the absorption peaks in the range of 1150–950 cm^−1^ may represent the –C–O–C– groups of saponin (Li et al. 2010).

From the SD-IR spectrum of VAE, the peaks at 1780 cm^−1^ and 1746 cm^−1^ are the absorption peaks of ester carbonyl C = O stretching, whereas the peaks at a higher wave number are the lactone carbonyl absorption peaks. Hence, VAE contains both lactones and esters. Also, the peaks present at 1738, 1706 (C = O stretching), 1619, 1605, 1518, 1497 (aromatic ring skeleton vibration), 1470, 1378 (C–H bending), and 1077 cm^−1^ (C–O stretching) in the SD-IR spectrum indicated that the extract contains flavonoids, while the absorption peak that appeared at 989 cm^−1^ represents the –C–O–C– group stretching of saponin.

In the 2D-correlation IR experiments, the perturbation introduced will change the intramolecular and intermolecular interactions of the sample. It will also affect the vibration frequency and coupling effect of each group of molecules. Through analysis of the changes of the spectra, we can get the relevant information of the intramolecular interactions and intermolecular interactions of the functional groups (Sun et al. 2010). We analyzed the synchronous spectra of VAE within the range of 1200–1800 cm^−1^ due to the characteristic intensity change within this region (Figure 1(c)). In the synchronous spectrum of the 2D-correlation IR, the diagonal peak is named as ‘auto peak’, which results from the autocorrelation of perturbation-induced dynamic fluctuations of the IR signals. It indicates the susceptibility of the corresponding absorbance bands to a given external perturbation. The cross peaks located at the off-diagonal position reveals the relative intensity variations of a pair of group vibrations corresponding to their frequencies. A positive cross peak (red/green area) represents the consistency of the population change, showing either simultaneous increase or decrease of the different groups under an external perturbation. The more coordinated the intensity changes, the stronger the cross peak is. In contrast, a negative cross peak (blue area) represents the coordinated changes of band intensities in the opposite directions (Li et al. 2014). The strongest auto peak in the synchronous two-dimensional correlation (2D-IR) spectra of VAE is located at 1382 cm^−1^ (C-N stretching), which is contributed by the effective components, alkaloids, and similar components in the extract. A weaker auto peak at 1735 cm^−1^ corresponded to the C = O stretching of ester. The results suggested that the effective components are very susceptible to thermal perturbation, while the ester is more stable.

### Possible vasorelaxant mechanisms of VAE

Blood vessels are capable of adapting to parameter changes including temperature, neurosignals, and mechanical forces that frequently occur in the ambient environment in order to produce constant and precise physiological action in animal bodies. The vascular tone is strictly regulated by both the vascular endothelium and vascular smooth muscles through stimulation from internal or external vasoactive compounds acting on the channels, receptors, or enzymes in the blood vessel (Jakala et al. 2009; Yildiz et al. 2013; Rameshrad et al. 2016). From the first line of screening, VAE exhibited vasorelaxation on endothelium-intact aortic rings. The vasorelaxant effect was significantly decreased in endothelium-denuded aortic rings but still ended up with high vasorelaxant effect. This means the vasorelaxant effect mediated by VAE is not solely endothelium dependent but also partially affected by endothelium-independent relaxant factors.

Two major endothelium-dependent relaxing factors (EDRFs), NO and PGI_2_, had been well characterized. In NO signalling cascade, the breakdown of l-arginine is catalyzed by endothelial nitric oxide synthase (eNOS) and NO is diffused through the vascular smooth muscle cells (VSMC) to activate sGC for the catalysis of guanosine triphosphate (GTP) into cGMP (Wang 2009; Louis 2011). A moderately high nonselective NO inhibitor, L-NAME (10 µM), was selected and used due to its high solubility compared to N_ω_-nitro-l-arginine (L-NNA). It highly reduced the relaxing effect of VAE, hence it was suggested that NO was involved in VAE-mediated vasorelaxant effect. However, the involvement of NO was further assured by using the sGC inhibitor, ODQ, and NO/cGMP soluble guanylyl cyclase (Damiani et al. 2003; Ameer et al. 2010; Jin et al. 2011), or more specifically, the cGMP-dependent inhibitor, MB (Mayer et al. 1993). The subsequent results further support the involvement of NO through the cascade of NO signalling pathway down to cGMP. Both the blockers had suppressed the vasorelaxant effect of VAE, but MB exerted higher inhibitory effect compared to ODQ. This could be explained with the inclination of VAE in employing the cGMP-dependent pathway to cause vasorelaxation down the NO signalling cascade. Furthermore, this phenomenon can be explained with that fact that only the crude VAE was used instead of single compound; therefore, there might be a variety of vasoactive components present and enhanced the vasorelaxant effect of VAE through multiple signalling pathways.

Other than NO, PGI_2_ also contributed as a major endothelium-derived relaxing factor (EDRF) in endothelium-intact conditions, and thus, the cyclooxygenases (COX) inhibitor, indomethacin, had been utilized for these studies. Originally, PGI_2_ is produced from the intermediate prostaglandin H_2_ catalyzed by prostacyclin synthase, while the intermediate prostaglandin is synthesized by COX. Most nonsteroidal anti-inflammatory drugs (NSAIDs) tend to inhibit the activity of both COX-1 and COX-2. Indomethacin, however, has a rapid and low-affinity reversible binding to COX upon application. In a time-dependent manner, the binding affinity to COX slowly increases (Smith et al. 2000; Dannhardt and Kiefer 2001). Therefore, indomethacin is frequently selected for these studies. Relatively low concentration of indomethacin dramatically suppressed the vasorelaxant effect of VAE. This suggested that PGI_2_ indirectly contributed to the vasorelaxant effect of the extract.

The muscarinic receptor (M_3_) is Gα_q_-protein-coupled receptor which is present in both the endothelium and the VSMC. However, the M_3_ receptors are much more dominant in the endothelium, and therefore, the effect of acetylcholine was significantly reduced in endothelium-denuded aortic rings. The activation of the M_3_ receptor would stimulate the phospholipase C (PLC) signalling pathway cascade, which upon activation would cause vasorelaxation in the endothelium but the opposite effect in VSMC. However, the nonselective inhibitor, atropine, was more frequently utilized with evidence of the presence of M_3_ receptor (Walch et al. 2000), even though there was insufficient information to prove this statement. Atropine tends to inhibit the vasorelaxant effect of VAE at the application of the starting concentration, but the maximum relaxation was still almost the same with the atropine-untreated group after the final cumulative concentration was applied. Apparently, VAE-mediated vasorelaxant effect was involved in the muscarinic receptor pathway from the beginning, but due to the limited magnitude of muscarinic receptors present in the isolated aorta rings within the organ bath, the vasorelaxant effect mediated by others receptors overcame the inhibition effect of the atropine on the muscarinic receptors. Therefore, the vasorelaxant effect increased after the final concentration was applied. The slight rightward shift of the graph suggested that VAE could act competitively on the muscarinic receptors with atropine. The binding of VAE to the receptors exceeded the binding of atropine due to the increasing concentration of VAE that was added simultaneously into the same organ bath. In addition, the reversible binding of atropine could be another factor that contributes to this result. Therefore, the result suggests that VAE had been employed by the muscarinic receptors when exerting vasorelaxation.

From the results shown, the removal of the endothelium highly attenuated the vasorelaxant effect of VAE, suggesting the involvement of the endothelium-independent relaxing factors such as β_2_-adrenoreceptor, which is a Gα_s_-protein-coupled receptor (GPCR) and is only present on the membrane of VSMC. The activation of this receptor will promote vasorelaxation by activating a cascade of signalling pathway through the activation of the GPCR, which will stimulate the activity of adenylyl cyclase (AC) to catalyze the breakdown of ATP to cAMP and subsequently causing vasorelaxation. Propranolol, the nonselective β_2_-adrenoreceptor inhibitor, was used most in similar studies due to its higher selectivity to the isolated tissue (Buch 2010). Propranolol showed a similar vasorelaxation trend as the muscarinic receptor mechanism. A slight rightward shift with similar maximum relaxation to the control group was observed, which suggested the competitive relationship between the antagonist propranolol and VAE acts on the same binding sites. The increased concentration of VAE overcame the inhibition effect of propranolol and produced an increasing rate of vasorelaxation. The results clearly showed that the endothelium-independent vasorelaxation effect exhibited by VAE belonged to the β_2_-adrenoreceptor pathway.

Other than endothelium-dependent factors, vascular tone is strictly regulated by the membrane potential, which is normally regulated via the correlation among different ion channels. The majority of them include the potassium and calcium channels. These channel-linked receptors react by allowing the inward or outward movements of ions through the membrane, which are widely distributed in both the endothelium and the VSMC (Nelson and Quayle 1995). Typically, four types of potassium channels were studied in this experiment. The K_Ca_, K_ir_, K_ATP_, and K_v_ were tested by using their respective antagonists. All the potassium channels function in regulating the electrochemical gradient in the cells by controlling the action potential. The activation of potassium channels can cause membrane hyperpolarization (Jakala et al. 2009; Yildiz et al. 2013) due to the outward flow of the potassium, thus inducing the closing of the calcium channels and leading to vasorelaxation. The activation of the potassium channels that occur in the endothelium could be attributed to the endothelium-derived hyperpolarizing factor (EDHF) (Luksha et al. 2009). From the results shown, the nonselective K_Ca_ blocker, TEA, and the K_ir_ blocker, BaCl_2_, significantly attenuated the vasorelaxing effect of VAE with similar magnitudes of reduction in terms of maximum relaxation percentage. The selective blocker of K_ATP_, which is glibenclamide, and the nonselective K_v_ blocker, 4-AP, contributed to a slight suppression on the maximum vasorelaxation of VAE. From the results shown, TEA exhibited the highest inhibition on vasorelaxation compared to other potassium channels, which suggested a high probability of involvement of the calcium channels since they were mediated by the K_Ca_ channel. However, a similar trend was observed during the earlier stage of antagonist applications in all four potassium channel studies. These trends showed a dramatic inhibition of relaxation during earlier cumulative concentrations of VAE applied and started to decline from the third to last concentrations (0.07 mg/mL until 2.55 mg/mL) of VAE applied. This rightward-shifted trend of concentration–response curves could be explained due to the competitive binding sites between vasoactive components of VAE and the antagonists. Hence, the vasorelaxation induced by VAE was suggested to be capable of mediating all four potassium channels.

Other than the potassium channels, the calcium ion movement plays a key role in vascular tone regulation through action potential. The activation of the VOCC causes the entry of extracellular calcium ions into the cytosol, which happens with increased extracellular potassium conductance as well as the depolarization of the VSMC membrane. This channel was well-characterized for calcium entry (McFadzean and Gibson 2002) and has been classified as a dihydropyridine-sensitive calcium channel (Feletou and Vanhoutte 2005). Therefore, the selective L-type calcium channel VOCC blocker, nifedipine, was used to test this mechanism. Relatively, low concentration of EGTA was used to deplete the calcium of the isolated aortic ring before pre-treatment of the antagonist and incubation with calcium-free buffer. The addition of calcium chloride in a cumulative concentration successfully elicited a concentration-dependent vasoconstriction response in control, positive, and experimental sets. In the set of positive controls, the contraction response was abolished almost completely by the high concentration of antagonist application. Results showed that the influx of extracellular calcium due to the addition of CaCl_2_ was partially inhibited (35% of inhibition) by a low concentration of VAE. Over 50% of contractile response was successfully inhibited upon the addition of a high concentration of VAE, which implied its concentration-dependent inhibition properties. Identical trends of contractile responses were exerted by VAE, with attenuated contractile responses occurring with the cumulative addition of 100 µM of CaCl_2_. These trends may have occurred due to the depolarization reaching a maximum point and the activation of the potassium channels for inducing hyperpolarizing conductance. The significant inhibition of VAE on the contractile response induced from the influx of extracellular calcium showed that the vasorelaxant effect of the extract exerted throughout the isolated aorta rings was abundantly mediated by VOCC. In this study, the intracellular calcium was washed out with EGTA, which ensures that the contraction of the aortic rings after pre-contraction by PE was caused only by the calcium released from the sarcoplasmic reticulum. VAE was able to reduce vasoconstriction evoked by PE in a concentration-dependent manner, by inhibiting the intracellular release of calcium into the cytosol through IP_3_ receptor.

## Conclusions

Based on the systematical analysis of VAE by using conventional FTIR, SD-IR, and 2D-correlation IR analysis, we confirmed the presence of alkaloids, flavonoids, and saponins within the extract. These compounds are likely to be the effective components that contribute to vasorelaxant activity. In conclusion, the crude VAE consists of multiple chemical components which could exhibit a strong vasorelaxant effect on isolated aortic rings due to its employment of multiple signalling pathways. The major route for inducing the vasorelaxant effect by VAE in *in vitro* isolated aortic rings was EDRFs which includes PGI_2_ and NO, followed by channel-linked receptors (as K^+^ channels opener), and subsequently M_3_- and β_2_-receptors signalling pathways. Since the experiment has only been assayed *in vitro* using aortic assay model, which may or may not be applicable to *in vivo*, therefore, antihypertensive effect of VAE can be performed using *in vivo* models. Moreover, further studies on VAE are required in order to identify the exact pure chemical compounds present that contribute to its major vasorelaxant effects.
